# Microstructural abnormalities in white and gray matter in obese adolescents with and without type 2 diabetes

**DOI:** 10.1016/j.nicl.2017.07.004

**Published:** 2017-07-05

**Authors:** Arie Nouwen, Alison Chambers, Magdalena Chechlacz, Suzanne Higgs, Jacqueline Blissett, Timothy G. Barrett, Harriet A. Allen

**Affiliations:** a School of Psychology, University of Birmingham, Birmingham, UK; b The Medical School, University of Birmingham, Birmingham, UK

**Keywords:** HbA1c, Haemoglobin A1c, Type 2 diabetes, Obesity, White matter, Gray matter, Demyelination

## Abstract

**Aims/hypotheses:**

In adults, type 2 diabetes and obesity have been associated with structural brain changes, even in the absence of dementia. Some evidence suggested similar changes in adolescents with type 2 diabetes but comparisons with a non-obese control group have been lacking. The aim of the current study was to examine differences in microstructure of gray and white matter between adolescents with type 2 diabetes, obese adolescents and healthy weight adolescents.

**Methods:**

Magnetic resonance imaging data were collected from 15 adolescents with type 2 diabetes, 21 obese adolescents and 22 healthy weight controls. Volumetric differences in the gray matter between the three groups were examined using voxel based morphology, while tract based spatial statistics was used to examine differences in the microstructure of the white matter.

**Results:**

Adolescents with type 2 diabetes and obese adolescents had reduced gray matter volume in the right hippocampus, left putamen and caudate, bilateral amygdala and left thalamus compared to healthy weight controls. Type 2 diabetes was also associated with significant regional changes in fractional anisotropy within the corpus callosum, fornix, left inferior fronto-occipital fasciculus, left uncinate, left internal and external capsule. Fractional anisotropy reductions within these tracts were explained by increased radial diffusivity, which may suggest demyelination of white matter tracts. Mean diffusivity and axial diffusivity did not differ between the groups.

**Conclusion/interpretation:**

Our data shows that adolescent obesity alone results in reduced gray matter volume and that adolescent type 2 diabetes is associated with both white and gray matter abnormalities.

## Highlights

• Type 2 diabetes and obesity in adolescents is associated with reduced gray matter volume.
• Type 2 diabetes was associated with significant regional changes in FA.
• FA reductions within these tracts were explained by increased RD.
• Mean diffusivity and axial diffusivity did not differ between the groups.

## 1 Introduction

There has been a marked world-wide increase in the prevalence of type 2 diabetes among young persons (Pinhas-Hamiel and Zeitler, 2005, Alberti et al., 2004). Although the cause is likely to be multi-factorial, childhood obesity is believed to be an important underlying factor (Pinhas-Hamiel and Zeitler, 2005, Haines et al., 2007).

Type 2 diabetes in adolescence is associated with structural brain abnormalities (Yau et al., 2010, Bruehl et al., 2011). Adolescents with type 2 diabetes are reported to have significantly reduced volume in hippocampus and prefrontal brain regions and higher rates of global cerebral atrophy compared to obese adolescents (Bruehl et al., 2011). These associations are similar to those reported for adults with type 2 diabetes (Moulton et al., 2015, Brundel et al., 2010, Anan et al., 2012, Hsu et al., 2012, Chen et al., 2012). For instance, gray matter reductions have been identified by Voxel Based Morphometry (VBM; a neuroimaging analysis technique using statistical parametric mapping), in adults with type 2 diabetes compared with healthy weight controls (Chen et al., 2012). Furthermore, differences in cortical white matter in adults with type 2 diabetes compared with healthy controls have also been found (Hsu et al., 2012, Chen et al., 2012).

In adults with type 2 diabetes, changes in gray matter volume have also been associated with levels of visceral fat (Anan et al., 2012) and several studies have found gray matter differences between obese patients and normal weight controls (Smucny et al., 2012, Pannacciulli et al., 2006). However, in adolescents, gray matter reduction was only observed in obese adolescents with type 2 diabetes and not those without diabetes, contrary to findings in adults, (Yau et al., 2010, Bruehl et al., 2011). Because cortical changes have been reported in obese adolescents without diabetes compared to healthy controls (Yokum et al., 2012) and in those with insulin resistance (Ursache et al., 2012), there is a need to compare between adolescents with type 2 diabetes, obesity and healthy weight.

A recent study comparing youth with type 2 diabetes to non-diabetic obese and healthy weight peers found that those with type 2 diabetes had reduced thalamic volume (Rofey et al., 2015). Although not statistically significant, the study also found microstructural white matter differences indexed by fractional anisotropy (FA) between the groups; those with type 2 diabetes showing the lowest FA levels (Hsu et al., 2012). However, the Rofey et al. (2015) study included only five participants per group and measured changes in a limited set of brain areas.

The exact extent and location of gray and white matter differences in adolescents with type 2 diabetes requires further examination using more accurate and impartial techniques, such as systematic voxel-wise mapping across the entire cortex and comparison across obese, type 2 diabetic and normal weight adolescents. The aim of this study was to examine whether type 2 diabetes and obesity are complementary or independent correlates of structural brain differences observed in adolescents with type 2 diabetes. Moreover, as FA is the combined measure of both radial and axial diffusion, which are often suggested to indicate demyelination and axonal degeneration respectively (Wozniak and Lim, 2006), we examined the composites of FA independently.

## 2 Methods

### 2.1 Participants

Fifteen adolescents with type 2 diabetes, 21 obese and 22 control adolescents participated (see Table 1a, Table 1b). All adolescents with type 2 diabetes were referred to the study by collaborating paediatric endocrinologists in the Midlands and North-West of England. Obese adolescents were either referred by dieticians or responded to study advertisements; control participants were recruited from local schools. Recruitment took place from November 2010–October 2012.

**Table 1a:** Demographic and clinical characteristics of the VBM groups. Participants with type 2 diabetes (T2DM) were referred to the study by Paediatric Endocrinologists and obese adolescents were either referred by dieticians or responded to study advertisements. Where possible data for insulin and diabetes related measures were also collected by the study team.

Characteristic	T2DM *N* = 14	Obese *N* = 20	Controls *N* = 19	F or Fisher's exact test	p
Age	16.1 ± 1.5	14.9 ± 2.00	16.4 ± 1.7	3.93	0.026
Sex (female, *n*, %)	14 (100%)	15 (75%)	14 (74%)		
Ethnicity (*n*)					
White	6	9	9	8.0	0.36
Asian	8	7	7		
Black	0	3	0		
Other	0	1	3		
SD-BMI (sd)	2.22 ± 1.55	3.25 ± 0.78	0.23 ± 0.96	38.08	< 0.0001
Fasting blood glucose (mmol/l) ± sd	9.54 ± 3.88 *n* = 11	4.9 ± 0.53 *n* = 20	4.77 ± 0.51 *n* = 17	26.32	< 0.0001
Fasting insulin (pmol/l) ± sd	257.7 ± 171.7 *n* = 6	172.8 ± 141.8 *n* = 19	77.2 ± 72.6 *n* = 16	5.30	0.009
HbA_1c_ (%) ± sd (mmol/mol)	8.10 ± 2.26 (65.0) *n* = 13	5.56 ± 0.39 (37.3) *n* = 19	5.35 ± 0.32 (35.0) *n* = 14	21.29	< 0.0001
HOMA-IR ± sd	102.2 ± 120.2 *n* = 5	40.0 ± 37.6 *n* = 19	16.5 ± 15.8 *n* = 16	5.97	0.006
c-Peptide (pmol/l) ± sd	1386.8 ± 905.6 *n* = 6	1226.7 ± 572.6 *n* = 18	762.7 ± 274.4 *n* = 16	4.37	0.020
2 h-OGTT (mmol/l) ± sd	NA	6.84 ± 1.49 *n* = 20	5.31 ± 0.97 *n* = 18	13.58	< 0.001
Duration of diabetes (months) ± sd	32.6 ± 30.0	NA	NA		
Range	7–106 (IQR = 36)				
Diabetes treatment (*n*)					
Metformin	6	4	NA		
Metformin + gliclazide	1	0			
Metformin + insulin	2	NA	NA		
Insulin	2	NA	NA		
GLP-1 agonist	1	0	NA		

Values are means ± SD; T2DM = type 2 diabetes; IQR = interquartile range; NA = not applicable.

**Table 1b:** Demographic and clinical characteristics of the TBSS groups.

Characteristic	T2DM *N* = 13	Obese *N* = 13	Controls *N* = 20	F or Fisher's exact test	p
Age	16.00 ± 1.6	15.0 ± 1.9	16.1 ± 1.9	1.34	0.27
Sex (female, *n*, %)	12 (100%)	10 (77%)	14 (70%)		
Ethnicity (*n*)					
White	5	8	10	7.03	0.54
Asian	7	3	7		
Black	0	1	0		
Other	0	1	3		
SD-BMI ± sd	2.01 ± 1.51 *n* = 13	3.11 ± 0.65 *n* = 13	0.32 ± 0.98 *n* = 20	27.54	< 0.0001
Fasting blood glucose (mmol/l) ± sd	8.87 ± 3.87 *n* = 9	4.95 ± 0.56 *n* = 13	4.78 ± 0.49 *n* = 19	16.91	< 0.0001
Fasting insulin (pmol/l) ± sd	227.5 ± 176.0 *n* = 7	178.2 ± 1.48.5 *n* = 13	72.2 ± 70.1 *n* = 18	5.08	0.012
HbA_1c_ (%) ± sd (mmol/mol)	7.80 ± 1.97 (61.7) *n* = 12	5.55 ± 0.39 (37.2) *n* = 13	5.29 ± 0.33 (34.3) *n* = 16	20.05	< 0.0001
HOMA-IR ± sd	102.2 ± 120.2 *n* = 5	41.5 ± 39.8 *n* = 13	15.4 ± 15.2 *n* = 18	6.11	0.006
c-peptide (pmol/l) ± sd	1265.86 ± 886.53 *n* = 7	1296.67 ± 525.51 *n* = 12	747.28 ± 264.98 *n* = 18	5.09	0.012
OGTT (mol/l) ± sd	NA	6.79 ± 1.77	5.38 ± 0.94	9.01	< 0.005
Duration of diabetes (months) ± sd	30.8 ± 23.0	NA	NA		
Diabetes treatment (*n*)					
Metformin	5	1	NA		
Metformin + gliclazide	1	0			
Metformin + insulin	2	NA	NA		
Metformin + gliclazide + insulin	1	NA	NA		
GLP-1	1	0	NA		

Values are means ± SD; T2DM = type 2 diabetes; NA = not applicable.

Selection criteria included: (1) aged between 12 and 18 years, (2) being able to understand and read English and (3) being diagnosed for at least 6 months (for the type 2 diabetes group). Each adolescent's BMI was converted to a Z score (SD-BMI) based on the British 1990 growth reference for height, weight, and body mass index (Cole et al., 1995). Obese adolescents were defined as having a SD-BMI exceeding 1.96 standard deviations from the mean (> 95th percentile). We excluded adolescents if they had (1) major medical conditions (other than type 2 diabetes, polycystic ovarian syndrome, hirsutism, which were included) or learning disabilities, (2) any contraindication to being in a MRI scanner or (3) major changes in diabetes related medication in the past 6 months. None of the adolescents in our study had diabetes complications.

Two participants were excluded due to movement artefacts, one due to signal loss and one due to a brain abnormality. T1 weighted scans (see below) were obtained from 14 participants with type 2 diabetes, 20 obese participants and 19 control group participants. Due to discomfort during scanning not all participants underwent a diffusion weighted scan. Hence, whole brain diffusion weighted scans (see below) were obtained from 12 adolescents with type 2 diabetes, 13 obese and 20 control participants. Fully informed consent was taken from all participants and their respective parent/guardian prior to participation. The study was carried out in accordance with Declaration of Helsinki for experiments involving humans and approved by the National Research Ethics Service and the Birmingham University Imaging Centre.

### 2.2 Procedure and measures

For all participants weight and height were measured wearing light clothing with footwear removed to calculate BMI. Prior to scanning, blood glucose was measured by the finger prick method, using a FreeStyle Optium Blood Glucose Monitor. To ensure control and obese participants did not have undiagnosed diabetes, their fasting glucose (Vitros 950 Dry Chemistry Analyser; Ortho Clinical Diagnostics, High Wycombe, UK), insulin by a nonspecific insulin ELISA method (Mercodia Iso-Insulin assay; Diagenics Ltd., Milton Keynes, UK), C-peptide, measured by ELISA (Dako, Ely, UK) at the Regional Endocrine Laboratory, University Hospital Birmingham (fasting reference ranges quoted from this laboratory are 200–800 pmol/l), and HbA1c (high-pressure liquid chromatography method) were measured and they underwent a 2-h oral glucose tolerance test. For type 2 diabetes patients these measures were obtained from the patient records. Duration of diabetes was also recorded. HOMA-IR, indicating insulin resistance, was calculated using the following formula: (fasting glucose × fasting insulin) / 22.5 (Matthews et al., 1985).

### 2.3 Image acquisition

MRI was performed on a Phillips 3 T scanner with 8-channel phased array SENSE head coil. A T1-weighted 3D image was acquired for each participant with TR 8.4 ms, TE 3.8 ms, flip angle 8, matrix resolution 288 × 288 and 175 sagittal slices with a voxel size of 1 × 1 × 1 mm. A subset of participants were scanned using a diffusion sequence employing echo planar imaging (75 slices with isotropic 2 × 2 × 2 mm^3^ voxels, TR = 9360 ms, TE = 77.8 ms). Diffusion data was acquired in 61 gradient directions with a b value of 1500 s/mm^2^, and 1 volume was acquired with no diffusion weighting (b = 0 image).

### 2.4 Image processing

We used VBM to examine structural differences in gray matter between the three groups. Pre-processing of the T1-weighted images was done using SPM8 (http://www.fil.ion.ucl.ac.uk/spm) and the VBM8 tool-box (http://dbm.neuro.uni-jena.de) in MATLAB v7.1 (MathWorks, Natick, MA, USA). The Template-O-Matic toolbox (Wilke et al., 2008) for SPM8 was used to generate an age and gender specific template in Montreal Neurological Institute (MNI) space for use with the VBM8 Toolbox. These tissue probability maps (TPM) were produced using the matched template approach with participant's age and gender as defining variables. This avoided using the standard adult reference data (Wilke et al., 2003) and introducing a systematic bias into the segmentation process, as has been previously demonstrated for paediatric MRI data (Raschle et al., 2011).

Within the same generative model (Ashburner and Friston, 2005) all T1-weighted images were segmented and the tissue segments normalized to the customised TPM using an affine transformation. These images were then used to create a study specific template using the Diffeomorphic Anatomical Registration Through Exponentiated Lie algebra (DARTEL) registration method (Ashburner, 2007).

All T1 weighted images were then segmented based on the previously estimated segmentation parameters, spatially normalized to the study specific template and corrected for bias-field inhomogeneities using the unified algorithm in VBM8 toolbox (Ashburner and Friston, 2005). Non-linear only normalization to the study specific template was used to account for individual brain sizes. The gray matter (GM) segmented images were then smoothed with 6 × 6 × 6 mm Gaussian kernel.

Between-group differences in GM volume were assessed in SPM8 using one-way ANOVA for three independent groups controlling for age and gender. For each contrast, statistical parametric maps were computed on a voxel by voxel basis to test for morphological differences between groups. In agreement with previous VBM studies for whole-brain analysis, clusters were considered significant at the threshold of p < 0.001, using an extent threshold of 200 contiguous voxels. This level is in agreement with published standards for applying VBM when there are priori hypotheses for regional differences (Ashburner et al., 2003).

Tract based spatial statistics (TBSS) were used to assess group differences in fractional anisotropy (FA), mean diffusivity (MD), axial diffusivity (AD) and radial diffusivity (RD). All diffusion data was analysed using FMRIB Software Library (FSL, Oxford, UK; Smith et al., 2004). First, the eddy current correction tool was used to align all diffusion volumes to the no diffusion volume using affine registration. A binary brain mask was created from the non-diffusion weighted image using the Brain Extraction Tool. Using DTIFit within the FSL FDT toolbox (Smith et al., 2004) diffusion tensor models were fitted for each voxel within the brain mask creating FA maps for each participant.

Using the TBSS pipeline (Smith et al., 2006) the most typical FA map in the sample was identified and all other participant FA images were aligned to it, then the entire aligned dataset was affine-transformed into 1 × 1 × 1 mm^3^ MNI152 space. The transformed FA images were averaged to create a mean FA image and thinned to produce a mean FA skeleton using a threshold value of 0.15 for the FA level. Each participant's FA map was then aligned onto the mean FA skeleton. Between-group differences in white matter were assessed using the FSL Randomise tool, which tests the t-value at each voxel location against a null distribution, comprised of 500 random permutations (v2.1; Anderson and Robinson, 2001). Statistical maps were corrected for multiple comparisons (p < 0.05) using threshold-free cluster enhancement (TFCE). Significant clusters were labelled with reference to JHU ICBM-DTI-81 and white-matter tractography atlases (Mori et al., 2005, Wakana et al., 2007, Hua et al., 2008).

Within regions identified as having reduced FA the individual contribution of AD and RD were explored. The second and third eigenvalues, l2 and l3, were averaged to provide a measure of RD and the first eigenvalue used as a measure of AD (Wozniak and Lim, 2006). Individual cortical maps of AD and RD values were created and (Smith et al., 2006) aligned onto the mean FA skeleton. For each participant the mean of AD and RD values located within regions of reduced FA, previously identified by the voxel-wise group analysis were calculated. Mean FA, MD, AD and RD values were compared among the three groups by conducting a one-way ANOVA followed by Bonferroni correction for multiple comparisons.

## 3 Results

### 3.1 Sample characteristics

As expected, participants with type 2 diabetes had significantly higher blood glucose, HbA1c, c-peptide and fasting insulin than controls and obese participants (see Table 1a, Table 1b). Six participants in the obese group met the WHO diagnostic criteria (World Health Organisation, 2006) of impaired glucose tolerance (IGT; fasting plasma glucose < 7.0 mmol/l and 2-h plasma glucose after OGTT of ≥ 7.8 and < 11.1 mmol/l); none of the obese participants had diabetes. There was a significant difference in age in the VBM sub-set, however age was controlled for in all analyses. Note that the analyses below were also performed without the male participants and results were compared to findings reported below. No qualitative differences were found, suggesting that gender effects do not drive our results.

### 3.2 VBM results

**Fig. 1:**
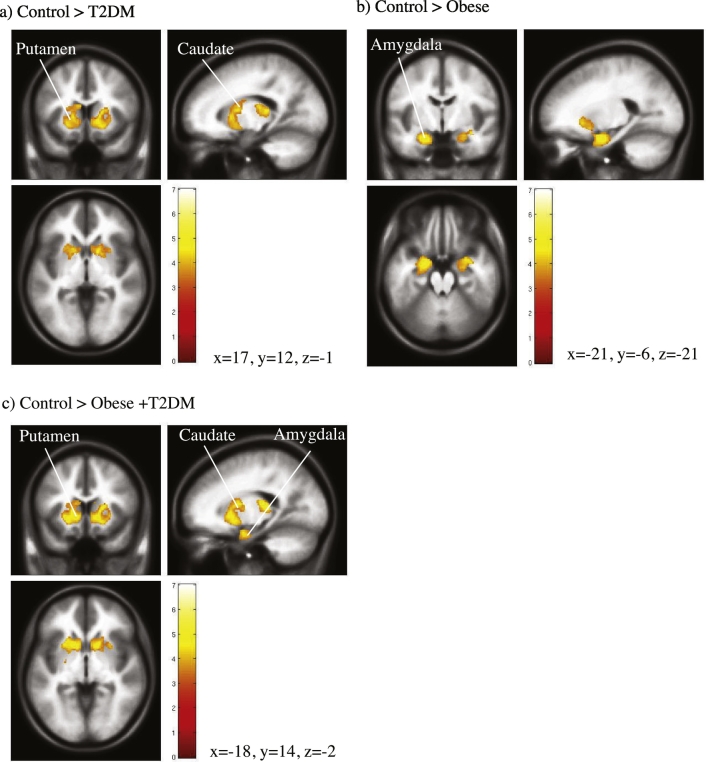
a–c. VBM analysis of GM volume. Areas in yellow show reduced GM volume in (a) type 2 diabetes adolescents than controls (MNI co-ordinates x = − 167, y = 11.8, z = − 0.7), (b) obese adolescents than controls (MNI co-ordinates x = − 21.4, y = − 5.6, z = − 20.7), (c) type 2 diabetes and obese adolescents than controls (MNI co-ordinates x = − 18, y = 13.8, z = − 2). Overlaid on an age and gender specific paediatric template created using TOM8 toolbox in SPM8. The left side of the image corresponds to the left hemisphere of the brain.

There were significant differences between GM volumes between the groups. Participants with type 2 diabetes showed smaller GM volume in the caudate and putamen bilaterally than the control participants (Fig. 1a, Table 2). The obese participants showed smaller GM volume in the right hippocampus, left putamen, left caudate and amygdala bilaterally than the control participants (Fig. 1b, Table 2). Controls had greater GM volume in putamen and caudate bilaterally, left amygdala and left thalamus than the other two groups together. (Fig. 1c, Table 2) and there were no areas where controls had lower GM volume than the other groups. There were no significant differences between obese and type 2 diabetes participants.

**Table 2:** Significant GM volume group differences.

Contrast	P FWE corr.	Cluster size (voxels^a^)	Z score	Co-ordinates			Anatomical location
				x	y	z	
Control > T2DM	< 0.001	1942	4.35	− 18	3	13	Left caudate
				− 10	2	− 14	Left putamen
				− 8	18	10	Left caudate
	< 0.001	1579	4.33	22	16	9	Right caudate
				14	10	− 2	Right putamen
Control > Obese	0.005	788	4.76	34	− 3	− 17	Right hippocampus
				26	− 7	− 20	Right hippocampus
				22	− 16	− 27	Right hippocampus/amygdala
	< 0.001	2106	4.63	− 18	− 1	− 23	Left amygdala
				− 14	14	− 2	Left putamen/caudate
				− 24	15	− 6	Left putamen
Control > Obese + T2DM	< 0.001	4327	4.73	22	18	7	Right putamen/caudate
				− 15	12	1	Left caudate
				− 24	14	− 6	Left putamen
	0.006	748	4.6	− 18	− 28	12	Left thalamus/caudate
				− 16	− 21	15	Left thalamus/caudate
	0.009	682	4.07	− 20	− 1	− 21	Left hippocampus/amygdala

a Voxel size = 1.5 × 1.5 × 1.5 mm; T2DM = type 2 diabetes.

To understand the relationship between lower GM volumes and the clinical variables, GM density values were extracted from each area showing difference (Suppl. Table 1). Bivariate correlations showed that lower GM density was associated with higher BMI in all regions showing significant differences in GM density between the control participants and the obese and T2DM participants with the exception of the right caudate (p > 0.09). After Bonferroni adjustment BMI remained significantly associated with GM density in the left caudate, left amygdala and left hippocampus. HbA1c correlated positively with GM density in the right hippocampus only, but not when only the control- and obese participants were considered (Suppl. Table 1) indicating, as expected, that group membership and HbA1c were not fully independent of each other. When considering only the control- and obese participants, the correlations between HOMA-IR and GM density in the left amygdala and hippocampus were significant (See Suppl. Fig. 1), although these were not significant when the diabetes group was included (after Bonferoni adjustment). When these variables were entered into a forward regression model while additionally controlling for age, it was found that BMI-SD was the only independent predictor of GM density explaining 42.3% of the variance in GM density in the amygdala (β = − 0.708; p < 0.0001) and 41.9% of the variance in GM density in the left hippocampus (β = − 0.706; p < 0.0001). Moreover, GM abnormalities were not more prominent in obese adolescents with impaired glucose tolerance but without type 2 diabetes (Wilks' λ = 0.487, *F*(7,11) = 1.66, p = 0.22). There were no significant correlations between GM density and duration of diabetes (all p's > 0.10).

### 3.3 TBSS results

**Fig. 2:**
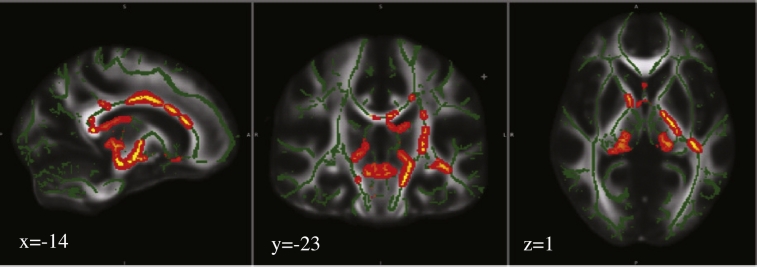
TBSS analysis of fractional anisotropy (FA) volumes. Areas in red are where FA values were significantly lower (p < 0.05, corrected by multiple comparison) in controls relative to type 2 diabetes adolescents. Areas of reduced FA (red) are thickened using the tbss_fill script implemented in FSL. Results are overlaid on the mean FA skeleton (green) and MNI152-FA template. The left side of the image corresponds to the right hemisphere of the brain. (For interpretation of the references to color in this figure legend, the reader is referred to the web version of this article.)

Compared to the controls, participants with type 2 diabetes had reduced FA in several brain areas (including the left corticospinal tract, corpus callosum, left fornix, left thalamic radiation, left retrolenticular internal capsule, inferior fronto-occipital fasciculus, right anterior corona radiata, left uncinate, left callosal body, cingulum, and left anterior external capsule) and no areas of higher FA (Fig. 2, Table 3). Overall, FA was negatively correlated with BMI (*r* = − 0.455; p = 0.002), and HOMA-IR (*r* = − 0.423; p < 0.0001), but HbA1c was not significant after Bonferroni adjustment correction (*r* = − 0.240; p = 0.032) (Suppl. Table 2). When BMI-SD, HbA1c and HOMA-IR were entered into a forward regression model while additionally controlling for age, it was found that HOMA-IR was the only independent predictor of FA explaining 8.1% of the variance in FA (β = − 0.370; p = 0.035) (See Suppl. Fig. 1).

**Table 3:** Regions of reduced FA in participants with type 2 diabetes compared to controls.

Cluster	Size (voxels^a^)	p	Peak MNI Co-ordinates			Region
			x	y	z	
1	2576	0.03	− 14	− 25	− 2	Left corticospinal tract
2	2085	0.03	− 5	− 2	26	Medial corpus callosum
3	611	0.05	− 8	− 1	− 16	Left fornix
4	557	0.04	22	− 34	0	Left thalamic radiation
5	422	0.04	− 34	− 28	1	Left retrolenticular internal capsule, left IFOF
6	134	0.05	15	21	23	Right anterior corona radiata, corpus callosum (genu)
7	74	0.05	− 12	10	− 17	Left uncinate
8	73	0.05	− 4	14	− 3	Left callosal body, cingulum
9	72	0.05	− 15	15	− 13	Left anterior external capsule, uncinate

a Voxel size = 1 × 1 × 1 mm.

**Fig. 3:**
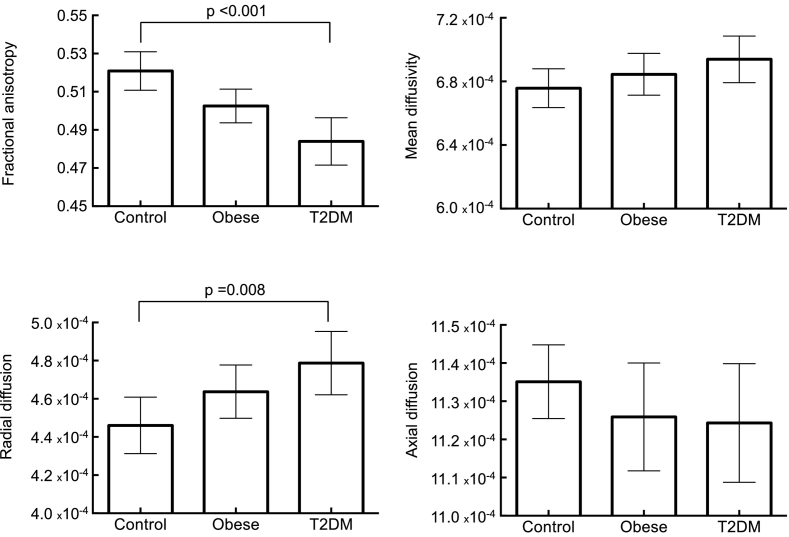
Mean FA, MD, RD, and AD values within regions of reduced FA. Error bars indicate 95% confidence intervals.

Regions of reduced FA in the type 2 diabetes group were mainly driven by a significant increase in radial diffusivity (p = 0.008 control vs. T2DM). The mean, radial or axial diffusivity of obese participants did not differ from the control or type 2 diabetes participants. Moreover, there was no difference in the mean diffusivity and axial diffusivity values between the three groups (Fig. 3). Radial diffusivity was positively correlated with BMI (*r* = 0.300; p = 0.045), HbA1c (*r* = 0.414; p = 0.008), and HOMA-IR (*r* = 0.341; p = 0.042), but when these variables were entered into a forward regression model while additionally controlling for age, none were independent predictors of radial diffusivity. WM abnormalities were not more prominent in obese adolescents with impaired glucose tolerance but without type 2 diabetes compared to their obese counterparts with normal glucose tolerance (Wilks' λ = 0.861, *F*(3,8) = 0.431, p = 0.737). Neither FA nor any of the diffusivity measures were significantly associated with the duration of diabetes (all p's > 0.38).

## 4 Discussion

Both adolescents with type 2 diabetes and obese adolescents had reduced GM volume compared with healthy weight adolescents in a range of medial brain regions. There was no difference in GM volume between the adolescents with type 2 diabetes and obesity. We also found reduced FA in adolescents with type 2 diabetes compared to the normal weight control participants in a range of central brain areas, and these reductions were best explained by increases in radial diffusivity.

To date, only two other studies examined structural brain abnormalities in adolescents with type 2 diabetes. Yau et al. (2010) found elevations in the apparent diffusion coefficient (ADC) in this group compared to weight-matched controls without diabetes, suggesting reductions in GM density. Based on manually drawn ROIs, consistent with the present study, lower GM volume has been found in the caudate and thalamus of adolescents with type 2 diabetes compared to normal weight adolescents (Bruehl et al., 2011) and GM levels of obese adolescents have been found to differ significantly with values of the control participants (Rofey et al., 2015). This study extends these findings to an evaluation of structural differences over the whole head.

Smaller hippocampal volumes and greater atrophy in frontal brain regions have been previously reported in obese adolescents with insulin resistance but without type 2 diabetes (Ursache et al., 2012) and this has also been found in adults with type 2 diabetes using focused region of interest analyses (Brundel et al., 2010, Gold et al., 2007), along with the basal ganglia (Moulton et al., 2015). In the present study, GM volume was reduced in the right hippocampus in the obese adolescents when compared to their healthy weight counterparts, but these reductions were not as manifest in our adolescents with type 2 diabetes. Neither did we find significant GM abnormalities in frontal brain regions in any of our groups. Hippocampal and frontal volume reductions seem more apparent when ROIs are being examined (Bruehl et al., 2011, Gold et al., 2007), but not in studies using the ADC (Yau et al., 2010) or a voxel by voxel approach, as in the present study. As ROI analyses are generally more sensitive to finding differences, these hippocampal GM abnormalities are likely to be small.

The data presented here suggest that reductions in GM volume in adolescents with obesity and in those with type 2 diabetes are primarily linked to higher BMI, characteristic of both conditions, rather than insulin resistance. However, given the small number of participants with both obesity and insulin resistance (n = 5) these results should be interpreted with caution.

Consistent with our findings, in terms of WM, lower FA values have also been found in other studies involving adolescents with type 2 diabetes (Yau et al., 2010, Rofey et al., 2015) and in most (Hsu et al., 2012, Hoogenboom et al., 2014, Zhang et al., 2014), but not all (van Bloemendaal et al., 2016) studies involving adults with type 2 diabetes. Although there is some overlap regarding the regions showing FA reduction (e.g. corona radiata), regions tend to differ across studies. Whereas Yau et al. (2010) and Hsu et al. (2012) found lower FA values in the frontal and temporal regions, in the current study and in Zhang et al. (2014) affected white matter tracts were mainly found in the central regions. Hoogenboom et al. (2014) reported lower FA values in the cingulum bundle and uncinate fasciculus, but analyses were limited to these specific regions of interest. The reasons for these regional variations in FA reductions are not clear but are likely to include differences in sample characteristics of both the diabetes and comparison groups and the relatively small sample sizes. These differences notwithstanding, the overall picture is that type 2 diabetes is associated with widespread reductions in FA, suggesting differences in white matter between the groups (Alexander et al., 2007).

FA is frequently used to refer to white matter ‘integrity’ but is only one of several indicators; we examined other diffusivity parameters to provide a more detailed picture of the underlying microstructural WM differences. Our data suggests that the reduction in FA in the adolescents with type 2 diabetes stemmed from an increased diffusivity in the radial axis, which has been associated with demyelination in animal studies (Song et al., 2002). Axial diffusivity, which has been associated with axonal damage or atrophy (Song et al., 2003, Concha et al., 2006), or mean diffusivity, indicating the average rate of water diffusion, did not differ between the groups. The present study is the first to report a detailed analysis of the WM microstructure in adolescents with type 2 diabetes and the pattern of results is generally consistent with that found in adults with type 2 diabetes (Hsu et al., 2012, Zhang et al., 2014, Reijmer et al., 2013). However, a study examining older people with type 2 diabetes found that changes in diffusivity appeared to be driven by changes in mean diffusivity in both the radial and axial direction (Reijmer et al., 2013b), whereas yet another study comparing obese adults with type 2 diabetes and lean normoglycaemic controls found reductions in axial diffusivity only (van Bloemendaal et al., 2016). Taken together, the literature seems to indicate that type 2 diabetes, especially at a younger age, is associated with increased radial, but not axial diffusivity, which is suggestive of demyelination rather than atrophy, but that with advancing age the risk of WM atrophy may also increase.

We did not find a difference in WM between participants with type 2 diabetes and obese participants or between obese and control participants. The relationship between obesity and WM abnormalities both in adolescents and adults has been the topic of several studies but, again, results are equivocal (Kullmann et al., 2015). Several studies found significant negative associations between BMI and FA reductions throughout the brain in young adults (Kullmann et al., 2015, Lou et al., 2014, Xu et al., 2013, Verstynen et al., 2013). Reduced FA in young obese adults was driven by increased radial and decreased axial diffusivity in one study (Verstynen et al., 2013), while another study found that BMI correlated with different diffusivity parameters in different areas of the brain, which may suggest that different underlying biological processes are involved in the relationship between BMI and reduced WM integrity (Xu et al., 2013). Furthermore, a recent study comparing age- and BMI-matched obese participants and lean participants found that obesity was associated with reduced axial diffusivity and reduced WM volume. BMI was the only independently factor associated with decreased WM integrity (van Bloemendaal et al., 2016), while yet another study found that obesity was associated with increased white matter density only in the striatum (Pannacciulli et al., 2006). Overall, these findings seem to suggest that the effects of obesity on the microstructure of WM are less specific than those associated with type 2 diabetes.

It is important to note though that the obese group in the present study included participants with impaired glucose tolerance, which may go some way in explaining that the WM structure of the obese group were in-between the diabetes group and control group. This is in line with Weinstein et al. (2015) who, in a study including young and middle-aged adults, found a dose-dependent relationship between brain integrity (defined by principle component analysis on FA and GM density measures) and categories of fasting blood glucose (normal glucose metabolism, impaired glucose metabolism and type 2 diabetes) with highest levels of brain integrity in people with normal glucose metabolism. However, in the current study WM abnormalities were not more prominent in obese adolescents with impaired glucose tolerance but without type 2 diabetes compared to their obese counterparts with normal glucose tolerance. It is of note that glucose tolerance is a biological continuum with artificial cut-offs to define normal glucose metabolism, impaired glucose tolerance and diabetes separated by small margins (7.8 mmol/l to 11.1 mmol/l for instance). As only six of the obese participants met the criteria for impaired glucose tolerance further studies with larger samples are needed to disentangle the effects of obesity, impaired glucose tolerance and type 2 diabetes on WM structure in young people.

The underlying mechanisms of the structural abnormalities in adolescents with type 2 diabetes are unknown and causal inferences cannot be made based on the present data, but a number of possible causes can be speculated. First, the GM and WM abnormalities in the brains of adolescents with type 2 diabetes or obesity could signify a delay in normal development. The normal development of the human brain is characterised by a steady decline in GM from adolescence into adulthood (Courchesne et al., 2012) along with an increase in WM (Lebel et al., 2012). These WM increases occur across the brain and are characterised by increased volume and FA, along with decreased mean diffusivity (MD) (Lebel et al., 2012). The pattern of results in the present study of reduced GM density along with reduced FA is the opposite to what would be predicted by delayed maturation of the brain. However, longitudinal studies are needed to examine this hypothesis.

Second, the results of our study suggest that the processes underlying the structural brain abnormalities in GM and WM in adolescents with type 2 diabetes may differ, with the GM changes primarily driven by factors associated with obesity and the WM changes are primarily driven by factors associated with insulin resistance. Obesity is linked to low-grade inflammation (Kumar and Cooke, 2002, Bastard et al., 2006) resulting in tissue degeneration (Veit et al., 2014). Insulin resistance has been associated with oxidative stress (Park et al., 2009), known to result, via multiple mediating pathways, in the production of free radicals (reactive oxygen species, ROS) causing damage to lipids, proteins and DNA, which in turn, has been found to result in nerve damage and segmental demyelination (Román-Pintos et al., 2016). It is important to note though that despite the relative strength in their association with GM and WM disturbances, obesity and insulin resistance are not independent of each other. Moreover, oxidative stress and inflammatory pathways are known to interact at multiple levels producing multiple outcomes (Sandireddy et al., 2014), which may explain some of the differences in findings across studies. Large studies are required to disentangle these interactions.

Inflammatory processes as a result of obstructive sleep apnea have also been associated with reduced FA and increased radial diffusivity in the absence of differences in mean and axial diffusivity (Chen et al., 2015). Although we did not monitor sleep apnea, it has been associated with obesity and type 2 diabetes in adolescents (Koren et al., 2015).

Others have found associations between decreased FA, mean diffusivity and systolic blood pressure (Maillard et al., 2012) in young adults. These associations were most notable in the anterior corpus callosum, an area similar to that found in the current study, suggesting that the WM abnormalities may be of vascular origins (Yau et al., 2013). Blood pressure data was not available for all diabetes participants in the current study but in contrast to Maillard et al. (2012) we did not find decreases in mean diffusivity in our study.

A strength of the current study is that we used objective automatic quantitative volumetric brain imaging techniques over the whole brain instead of focussing on a-priori set brain regions. Although this may have reduced the power of finding differences between the groups compared to ROI analyses, our findings are likely to be robust to find differences in FA between healthy weight adolescents and those with type 2 diabetes. This is the first study to use a study specific brain template to examine WM and GM abnormalities comparing adolescents with type 2 diabetes, those with obesity and healthy weight with objective techniques such as VBM and TBSS. Finally, participants came from clinics and schools from a large geographical region and major ethnic and social groupings were represented.

However, our study also has limitations. First, the sample sizes were relatively small for a structural study, which may have reduced the power of finding WM differences between the obese group and the type 2 diabetes groups and between the obese and control groups. Second, the current study did not include assessment of cognitive function. Type 2 diabetes has been associated with mild neuropsychological disturbances in older (Reijmer et al., 2013, Zhang et al., 2014) and younger people (Yau et al., 2010). Reduced myelin results in reduced processing speed, which, in turn, may affect cognitive function. However, all our participants were attending secondary schools or college at the appropriate level for their age suggesting that if cognitive deficits were present, they were likely to be mild to very mild. Future studies should examine whether the abnormalities in WM and GM found in this study result in reduced cognitive abilities. Third, as discussed above, the obese group in the present study included participants with impaired glucose tolerance, which may have affected the WM results.

## 5 Conclusions

Type 2 diabetes, even in adolescents, is associated with differences in white matter microstructure as indicated by lower FA in a large number of commissural, association and projection pathways. FA reductions were explained by increases in radial diffusivity, consistent with demyelination of white matter tracts. Compared to healthy weight controls, adolescents with type 2 diabetes and those with obesity also show reduced GM. It is currently unknown whether the abnormalities in GM and WM in adolescents with type 2 diabetes or obesity are reversible. Given the potentially serious consequences for these young people, longitudinal studies are now needed.

The following are the supplementary data related to this article.

Supplementary tables Image 1

Supplementary Fig. 1a–f Associations between clinical variables (BMI-sd, HbA1c, HOMA-IR) and left amygdala and hippocampus by (sub)group. Supplementary Fig. 1

## Funding

This work the study was made possible by a grant from the European Foundation for the Study of Diabetes/Novo Nordisk European Clinical Research Programme in Adolescents with Type 2 diabetes mellitus.

## Duality of interest

The authors declare that there is no duality of interest associated with this manuscript.

## Contribution statement

AN, HA, SH, JB, TB conceived and designed the study, contributed to data analysis and interpretation; AC and MC performed data analysis and interpretation, MC and HA made substantial contributions to data interpretation, and reviewed and revised the manuscript; SH, JB and TB reviewed and revised the manuscript; AN wrote and revised the manuscript and had overall responsibility for the integrity of the work as a whole. All authors approved the final version of the article.
