# Supplementary material for: Microstructural abnormalities in white and gray matter in obese adolescents with and without type 2 diabetes
Source: Neuroimage Clin. 2017 Jul 5;16:43–51. doi: 10.1016/j.nicl.2017.07.004 (PMC5514690; doi:10.1016/j.nicl.2017.07.004)
Supplement: Supplementary file 1 — Supplementary tables [file mmc1.docx]

Supplementary Table 1. VBM GM density correlations with clinical variables BMI, HbA1c and HOMA-IR.

|  | Left Caudate | Right Caudate | Right  Hippocampus | | Left  Amygdala | Right Putamen | | Left Thalamus | | Left  Hippocampus | |
| --- | --- | --- | --- | --- | --- | --- | --- | --- | --- | --- | --- |
| T2DM, Obese and Controls | | | | | | | | | | | |
| BMI SD | -0.411  (-.561 to -.260)  *p* = .002* | -0.232  (-.405 to -.063)  *p* = .095 | -0.398  (-.577 to -.199)  *p* = .003 | | -0.551  (-.712 to -.353)  *p* = <.0001* | | -0.291  (-.465 to -.109)  *p* = .035 | | -0.355  (-.587 to -.097)  *p* = .009 | | -0.551  (-.709 to -.352)  *p* = <.0001* |
| HbA1c | -0.006  (-.203 to .194)  *p* = .95 | -0.048  (-.228 to .131)  *p* = .65 | 0.272  (.100 to .468)  *p* = .009 | | 0.028  (-.158 to .214)  *p* = .79 | | -0.003  (-.183 to .184)  *p* = .98 | | -0.040  (-.221 to .140)  *p* = .70 | | 0.046  (-.135 to .238)  *p* = .66 |
| HOMA-IR | -0.169  (-.359 to .038)  *p* = .12 | -0.018  (-.197 to .141)  *p* = .87 | -0.023  (-.242 to .186)  *p* = .83 | | -0.290  (-.482 to -.092)  *p* = .008 | | -0.055  (-.246 to .120)  *p* = .62 | | 0.095  (-.293 to .089)  *p* = .40 | | -0.300  (-.494 to -.108)  *p* = .006 |
| Obese and Controls without T2DM | | | | | | | | | | | |
| BMI SD | -.430  (-.589 to -.301)  *p* = .006 | -.266  (-.441 to -.127)  *p* = .10 | | -.560  (-.737 to -.335)  *p* < .0001* | -.686  (-.834 to -.504)  *p* < .0001* | | -.315  (-.467 to -.189)  *p* = .05 | | -.339  (-.558 to -.111)  *p* = .035 | -.687  (-.828 to -.511)  *p* < .0001* | |
| HbA1c | 0.008  (-.252 to .219)  *p* = .95 | -0.040  (-.277 to .145)  *p* = .75 | | -0.088  (-.345 to .166)  *p* = .49 | -0.275  (-.529 to .002)  *p* = .031 | | 0.014  (-.222 to .217)  *p* = .91 | | -0.076  (-.305 to .149)  *p* = .55 | -0.267  (-.522 to .003)  *p* = .036 | |
| HOMA-IR | 0.224  (-.448 to .002)  *p* = .059 | -0.025  (-.219 to .157)  *p* = .83 | | -0.146  (-.372 to .101)  *p* = .22 | -0.399  (-.603 to -.178)  *p* = .001* | | -0.054  (-.284 to .147)  *p* = .65 | | 0.140  (-.359 to .066)  *p* = .24 | -0.409  (-.618 to -.180)  *p* = .001* | |

Note: Values are Pearson (BMI-SD) or Kendall’s tau (HbA1c, HOMA-IR) correlation coefficients (95%CI) and *p-*value*.* Bias corrected and accelerated confidence intervals were computed using bootstrapping (1000x) stratified by group.

*Statistically significant after Bonferroni adjustment (adjusted  *p-*level .05/21=.238).

Supplementary Table 2. TBSS associations of FA, RD, AD, MD and clinical variables BMI, HbA1c and HOMA-IR

|  | Mean FA | Mean RD | Mean AD | Mean MD |
| --- | --- | --- | --- | --- |
| T2DM, Obese and Controls | | | | |
| BMI SD | -0.455  (-.606 to -.286)  *p* = .002* | 0.300  (.014 to .541)  *p* = .045 | -0.225  (-.416 to -.033)  *p* = .14 | 0.179  (-.090 to .432)  *p* = .24 |
| HbA1c | -0.240  (-.433 to -.077)  *p* = .032 | 0.267  (.001 to .538)  *p* = .017 | -.031  (-.261 to .194)  *p* = .78 | 0.181  (-.412 to .058)  *p* = .11 |
| HOMA-IR | -0.423  (-.552 to -.305)  *p* < .0001* | 0.337  (.133 to .527)  *p* = .004* | -0.172  (-.047 to -.416)  *p* = .14 | 0.194  (-.047 to -.416)  *p* = .097 |
| Obese and Controls without T2DM | | | | |
| BMI SD | -0.397  (-.657 to -.094)  *p* =.022 | 0.272  (-.075 to .606)  *p* =.13 | -0.213  (-.496 to .074)  *p* =.23 | 0.155  (-.168 to .488)  *p* < .39 |
| HbA1c | 0.061  (-.196 to .315)  *p* =.65 | -0.020  (-.352 to .300)  *p* =.88 | -0.200  (-.471 to .069)  *p* =.14 | -0.113  (-.426 to .196)  *p* =.41 |
| HOMA-IR | -0.347  (-.540 to -.157)  *p* < .006 | 0.274  (.071 to .473)  *p* =.03 | -0.127  (-.412 to .169)  *p* =.32 | 0.144  (-.027 to .400)  *p* =.26 |

Note: Values are Pearson (BMI-SD) or Kendall’s tau (HbA1c, HOMA-IR) correlation coefficients (95%CI) and *p-*value*.*

*Statistically significant after Bonferroni adjustment (adjusted *p*-level .05/12=.0042).
