# Supplementary material for: Microstructural abnormalities in white and gray matter in obese adolescents with and without type 2 diabetes
Source: Neuroimage Clin. 2017 Jul 5;16:43–51. doi: 10.1016/j.nicl.2017.07.004 (PMC5514690; doi:10.1016/j.nicl.2017.07.004)
Supplement: Supplementary Fig. 1a–f — Associations between clinical variables (BMI-sd, HbA1c, HOMA-IR) and left amygdala and hippocampus by (sub)group. [file mmc2.docx]

Suppl. Figure 1a-f. Associations between clinical variables (BMI-SD, HbA1c, HOMA-IR) and left amygdala and hippocampus by (sub)group.

a.

b.

c

d

e

f
